# Identification of amino acid residues in protein SRP72 required for binding to a kinked 5e motif of the human signal recognition particle RNA

**DOI:** 10.1186/1471-2199-11-83

**Published:** 2010-11-13

**Authors:** Elena Iakhiaeva, Alexei Iakhiaev, Christian Zwieb

**Affiliations:** 1 Department of Molecular Biology, University of Texas Health Science Center at Tyler, 11937 US Highway 271, Tyler, Texas 75708, USA; 2 Texas College, Natural Sciences, 2404 North Grand Avenue, Tyler, TX 75702, USA

## Abstract

**Background:**

Human cells depend critically on the signal recognition particle (SRP) for the sorting and delivery of their proteins. The SRP is a ribonucleoprotein complex which binds to signal sequences of secretory polypeptides as they emerge from the ribosome. Among the six proteins of the eukaryotic SRP, the largest protein, SRP72, is essential for protein targeting and possesses a poorly characterized RNA binding domain.

**Results:**

We delineated the minimal region of SRP72 capable of forming a stable complex with an SRP RNA fragment. The region encompassed residues 545 to 585 of the full-length human SRP72 and contained a lysine-rich cluster (KKKKKKKKGK) at postions 552 to 561 as well as a conserved Pfam motif with the sequence PDPXRWLPXXER at positions 572 to 583. We demonstrated by site-directed mutagenesis that both regions participated in the formation of a complex with the RNA. In agreement with biochemical data and results from chymotryptic digestion experiments, molecular modeling of SRP72 implied that the invariant W577 was located inside the predicted structure of an RNA binding domain. The 11-nucleotide 5e motif contained within the SRP RNA fragment was shown by comparative electrophoresis on native polyacrylamide gels to conform to an RNA kink-turn. The model of the complex suggested that the conserved A240 of the K-turn, previously identified as being essential for the binding to SRP72, could protrude into a groove of the SRP72 RNA binding domain, similar but not identical to how other K-turn recognizing proteins interact with RNA.

**Conclusions:**

The results from the presented experiments provided insights into the molecular details of a functionally important and structurally interesting RNA-protein interaction. A model for how a ligand binding pocket of SRP72 can accommodate a new RNA K-turn in the 5e region of the eukaryotic SRP RNA is proposed.

## Background

The signal recognition particle (SRP) participates in the vital compartmentalization of every cell by guiding proteins towards their membrane translocation sites. Except in chloroplasts [1], SRP is a ribonucleoprotein which is composed of the SRP RNA and at least one protein, the exceptionally conserved SRP54. SRP interacts with secretory signal or membrane-anchor sequences as they emerge from the ribosomal exit tunnel and delays or blocks the translation of the to-be-targeted polypeptides. Subsequently, the SRP-ribosome nascent chain (RNC) complex binds to a subunit of the membrane-associated SRP receptor (SRα). SRP54 and SRα each recruit a guanosine triphosphate molecule and, upon the hydrolysis of both GTPs, the synchronized release of the signal sequence promotes the secretory protein to enter a protein-conducting channel (PCC) of the ER. The SRP returns to its free cytosolic state and initiates another protein targeting cycle [2-6].

**Figure 1:**
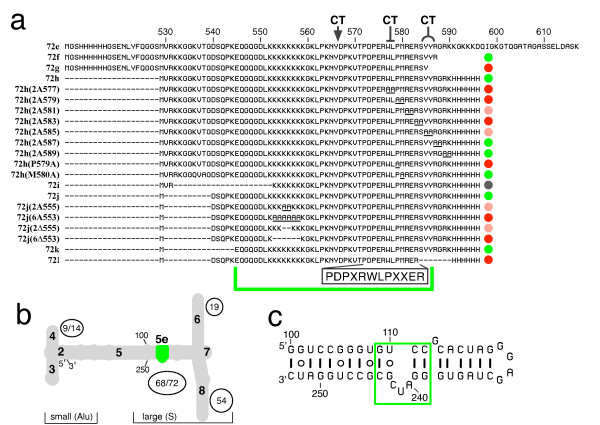
**Participants in the interaction between protein SRP72 and the SRP RNA**. a. Wildtype and mutant human SRP72 polypeptide fragments used for investigating their potential to interact with the 5e SRP RNA. Their names are given on the left. Amino acid residue numbering is according to the full-length human SRP72. Indicated are the mutated residues (underlined) and their effects on RNA-binding are shown by the colored dots. Green is for binding activities similar to the 72 h or 72 k fragments, red for no or weak binding, and pink for intermediate activities. The gray circle indicates that mutant polypeptide 72 i was prone to degradation. Potential cutting sites for Chymotrypsin (CT) are shown on top. The RNA binding region with its PDPXRWLPXXER motif (boxed) is indicated by the green brace. b. Outline of the human SRP. The RNA helices in the secondary structure (gray) are numbered from two to eight. The 5' and 3' ends and the two SRP domains are indicated, and the SRP proteins (circles and ovals) are positioned accordingly. The location of the 5e motif is shown in green as are the residue positions 100 and 240 of a neighboring loop. c. Predicted secondary structure of the 5e RNA. Numbering is according to full-length human SRP RNA excluding the helix-closing tetraloop (gray). The 11-nucleotide 5e motif is shown boxed.

The mammalian SRP is composed of an 303-nucleotide SRP RNA and proteins SRP9/14, SRP19, SRP54 and SRP68/72 [7]. Responsible for the translation delay are SRP9/14 and the terminal SRP RNA regions which form the small domain (Alu) of the elongated dumbbell shaped SRP. The large or S-domain participates in GTP-hydrolysis and signal peptide binding and release. It is composed of proteins SRP19, SRP54, and SRP68/72 bound to SRP RNA helices 5, 6 and 8 (see Figure 1b) [8-10]. Protein SRP72 was shown to form an unusually stable heterodimer with SRP68 [11,12] but also to bind to the SRP RNA independently of SRP68 or other components [13].

Cryo-electron microscopy combined with site-directed mutagenesis and footprinting experiments demonstrated that SRP68 possesses numerous contacts with the SRP RNA of the large SRP domain [12,14]. In contrast, SRP72 bound to a relatively small section of helix 5 which included the 11-nucleotide 5e motif, present in the larger SRP RNAs and composed of four symmetrically arranged base pairs interrupted by a three-nucleotide loop [15]. In eukaryotes, the first nucleotide of the loop (A240 in human SRP RNA) proved to be critical for the binding of protein SRP72 [16].

Several high-resolution structures of substantial portions of the SRPs from a variety of taxa have been solved and this knowledge contributed significantly to our understanding of how the SRP assembles and fulfills a multitude of functions [9,17-19]. Despite their vital importance for SRP assembly and essential contributions to the targeting of secretory polypeptides to the ER [20] detailed structural information about the SRP68 and SRP72 proteins is still lacking.

The focus of the presented work is human protein SRP72, the largest of the SRP proteins (74.6 kDa), and its association with the SRP RNA. Full-length SRP72 as well as a fragment of SRP72 (72 d, residues 531 to 659) were shown to bind independently and specifically to human SRP RNA with a K'a of 2.9 × 10^7 ^M^-1^. The RNA-binding SRP72 fragments shared a conserved region with the consensus sequence PDPXRWLPXXER at positions 572 to 583 (X is for any amino acid residue) and a cluster of positively charged amino acid residues at positions 552 to 559 [13]. We used site-directed mutagenesis and enzymatic structure probing to investigate the interaction between human SRP72 and the 5e SRP RNA motif. The data supported a three-dimensional model in which the 5e motif is at the center of a new RNA K-turn which engages the lysine-rich loop of SRP72 as well as certain amino acid residues from the PDPXRWLPXXER Pfam motif.

## Results and Discussion

### The minimal SRP RNA binding domain of human SRP72

Human SRP72, composed of 671 amino acids, had a molecular mass of 74,602 dalton, a pKi of 10.01, and 98.8% identity to its canine homologue [13]. The protein was shown previously to bind independently and specifically to the SRP RNA [13]. A fragment of SRP72 (72e, residues 531 to 617 of the complete human SRP72, Figure 1) also formed complexes with the full-length SRP RNA as well as with RNA fragments derived from the large domain of the SRP [16].

**Figure 2:**
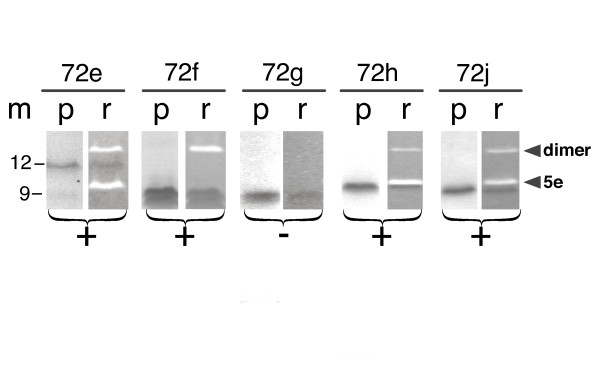
**5e RNA binding activities of polypeptides 72 e, 72 f, 72 g, 72 h and 72 j**. Purified his-tagged polypeptides 72 e to 72 j were incubated with in vitro transcribed 5e SRP RNA and Ni-NTA magnetic agarose beads as described in the Methods. The bound protein and RNA were analyzed by SDS PAGE followed by staining of the same gel with Coomassie blue (lanes labeled p) and Ethidium bromide (lanes labeled r). Molecular mass markers in kDa are shown in lane m. Plus signs indicate the formation of complexes, the minus sign below 72 g indicates that this polypeptide was unable to bind. Variable amounts of a material which probably represented 5e SRP RNA dimers were observed (arrow heads).

**Figure 3:**
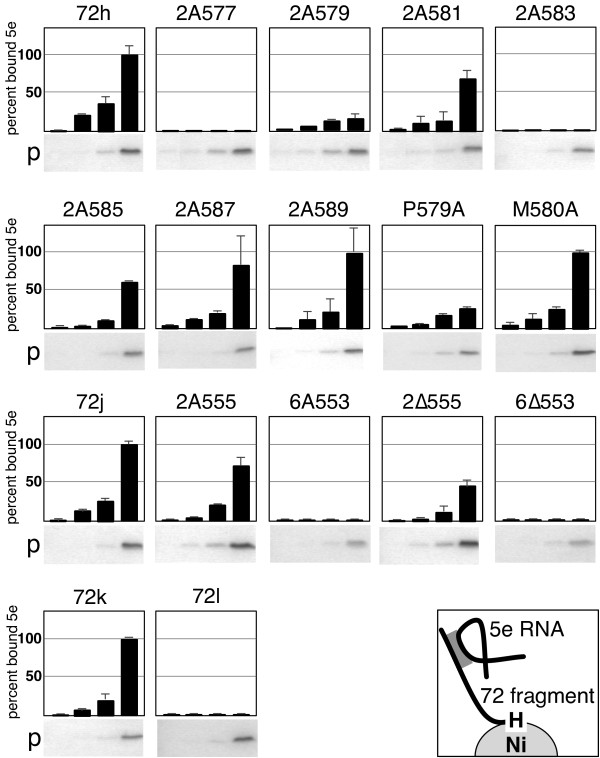
**5e SRP RNA binding activities of mutated SRP72 fragments**. For each construct, 82.5 ng of in vitro transcribed 5e SRP RNA was incubated with purified protein at the concentrations 0.056, 0.18, 0.56, and 1.8 uM. Samples were incubated with Ni-NTA magnetic agarose beads as described in the Methods. The bound protein and RNA were analyzed by SDS PAGE followed by staining with Coomassie blue (labeled p below each panel) and Ethidium bromide. The amount of bound RNA was measured and normalized to 100 percent in reference to the binding observed with 72 h, 72 j, or 72 k. The insert on the bottom right depicts the design of the assay.

Purified polypeptide 72e was used as the substrate for the initial site-directed mutagenesis experiments designed to identify the smallest polypeptide with undiminished RNA binding activity. The RNA ligand was a 46-nucleotide hairpin which contained the 5e motif (Figure 1c). Recombinant polypeptides were purified to homogeneity using affinity column (or beads) based approaches as described in Methods. Ni-NTA magnetic agarose bead were used to monitor the formation of complexes between his-tagged 72e or its purified mutant derivatives and the in vitro transcribed 5e RNA. Molecules which co-eluted from the beads in a buffer containing 250 mM Imidazole were analyzed by SDS PAGE and stained with Coomassie blue and Ethidium bromide (see Methods). The results shown in Figure 2 demonstrated that three polypeptides lacking several amino acid resides from the termini of 72e, namely 72f, 72h, and 72j, still were capable to form complexes with the 5e RNA. In contrast, 72g which was two amino acid residues shorter than the active 72f was unable to bind. Because previous experiments had shown that a chymotryptic cleavage at Y586 retained the ability to form a complex with the RNA, the C-terminus of the minimal active SRP72 RNA binding site appeared to include both tyrosines at positions 585 and 586 whereas R587 was dispensable [13]. The RNA binding activity of 72j suggested that, towards the N-terminus, the residues at positions 531 to 540 were not required. As was discovered later, removal of five additional residues in 72k (Figure 3) was also tolerated indicating that the N-terminus of the RNA binding region was located at or close to residue E545. Polypeptide 72i, shortened by six N-terminal residues when compared to 72k was expressed but subject to extensive proteolytic digestion and therefore not amenable to investigation (not shown). Variable amounts of Ethidium bromide-stained material with a molecular mass larger than that of the 5e RNA were seen on the gel and likely represented dimeric forms of 5e. The dimers lacked a hairpin loop but contained two tandem 5e motifs and were expected to bind to 72e as was indeed observed (Figure 2).

### Site-directed mutagenesis of the C-terminal region and the cluster of lysine residues

A recently updated alignment of 113 eukaryotic SRP72 sequences [21] confirmed the presence of two previously identified prominent features within the SRP RNA binding region of SRP72. The first corresponded to a lysine-rich region, KKKKKKKKGK, at positions 552 to 561 of the human protein. The second region was a previously discovered Pfam motif (PF08492), unique to SRP72, with the consensus sequence PDPXRWLPXXER at residues 572 to 583 (Figure 1). The distinctive properties of these regions prompted us to investigate by site-directed mutagenesis their contributions to the binding of SRP72 to the SRP RNA.

As substrate for mutagenesis we used the coding region of 72h which exhibited optimal binding to the 5e RNA (Figure 2). Standard molecular biology techniques, including PCR, ligation of annealed synthetic oligonucleotides, and engineered restriction sites were employed to generate plasmids which encoded the mutated his-tagged proteins. The his-tagged polypeptides were expressed in *Escherichia coli*, purified by adsorption on Ni-NTA beads, and the eluates were incubated with in vitro transcribed 5e RNA. Binding was measured by incubating fixed amounts of RNA with increasing concentrations of the purified wild-type or mutant polypeptides. The proteins and RNAs eluted from the Ni-NTA magnetic agarose beads were analyzed by SDS PAGE followed by staining with Coomassie blue and Ethidium bromide (see Methods). The results shown in Figure 3 demonstrated that all polypeptides bound to the magnetic beads because of the functional his-tag. No binding of RNA to the beads occurred at the lowest polypeptide concentrations used. Depending on the nature of the mutations, some peptides associated with the 5e RNA while others were unable to form complexes.

Two of the seven constructs with di-alanine changes in the C-terminal region, including the mutant polypeptides with changes of 577-WL-578 and 583-RS-585 in the Pfam motif, were inactive. Diminished binding to the 5e RNA was observed with alterations of 579-PM-580 (14 ± 9%) and 581-RE-582 (69 ± 14%). Interestingly, the mutation of the evolutionary conserved P579 within PDPXRWLPXXER to an alanine substantially reduced the binding activity (26 ± 1%). At the same time, the mutation at the variable M580 allowed the formation of complexes at wild-type levels (100 ± 4%).

The alanine changes at 585-YY-586 caused a significant reduction (60 ± 3%) in the ability to form complexes. Similarly, the deletion of seven amino acid residues, including 585-YY-586 and S584, in 72l yielded inactive polypeptides. Together with the dissimilar RNA binding activities of 72f and 72g (Figure 2) these results suggested an importance of Y586 for RNA binding. Examination of the SRP72 alignment showed that this tyrosine was only moderately preserved but changed conservatively to another aromatic residue most frequently a phenylalanine. Changes of 587-RG-588 and 589-RK-590 elicited formation of complexes near the levels observed with wild-type 72h (Figure 3). These results were consistent not only with the finding that 72f was able to bind to the RNA but also with results obtained previously using proteolytically digested SRP72 fragments [13].

When changing or deleting two residues near the center of the cluster of lysine residues, the ability to form complexes was significantly reduced (72 ± 14% for the 555-KK-556 change and 46 ± 12% for the matching deletion). Changing or removing six lysine residues abolished binding completely. These data suggested that the lysine-rich region contributed substantially to the RNA binding activity of SRP72.

### Chymotryptic accessibility of the SRP72 RNA binding domain

**Figure 4:**
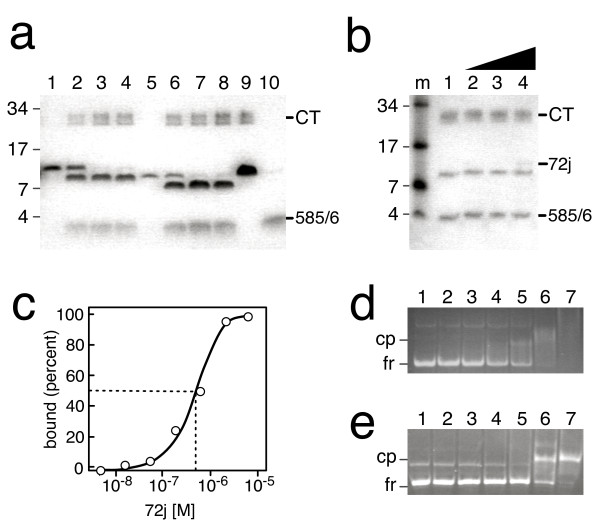
**Chymotryptic protection in the RNA binding domain of SRP72**. a. Digestion of purified 72 j (lane 1) with Chymotrypsin for 3, 6 or 10 minutes (lanes 2 to 4). Similarly, digestion of purified 72 k (lane 5) for 3, 6 or 10 minutes (lanes 6 to 8). Lane 9, the 72 l fragment (lacking residues 584 to 590) treated with Chymotrypsin for 10 minutes; lane 10, the 585/6 polypeptide, generated by a 10 minute chymotryptic digest of 72 k, retained on Ni-NTA magnetic agarose beads (see Methods for details). All samples were analyzed by electrophoresis on 12.5 percent SDS polyacrylamide gels followed by staining of the polypeptides with Coomassie blue. Molecular mass markers in kDa are indicated on the left and the migrations of Chymotrypsin (CT) and the his-tagged C-terminal 585/6 fragment are marked on the right. b. Digestion of 1.2 μg 72 j with Chymotrypsin in the absence of RNA (lane 1) or in the presence of increasing amounts of human Δ35 RNA (lanes 2 to 4)[23]. Indicated are molecular mass markers and the migrations of Chymotrypsin (CT), the 72 j polypeptide, and the his-tagged oligopeptide generated by the cleavages of the Tyrosines 585 or 586. c. Binding of human Δ35 to increasing amounts of 72 j. d. Results from gelshift experiment for the binding data shown in Figure 5c. Indicated are the mobilities of the free Δ35 RNA (fr) and its complex with 72 j (cp). Lane numbers correspond to the seven data points shown in Figure 5c. e. Similar to Figure 5 d, showing the formation of a complex between the 72 j and the 5e SRP RNA.

Chymotrypsin was shown to preferentially cleave peptides at trptophane, tyrosine and phenyalanine residues [22]. This presented an opportunity to probe the structure of the minimal SRP72 RNA binding region and the accessibilities of Y566, W571, and 585-YY-586. Polypeptides 72 j and 72 k were treated with a fixed amount of Chymotrypsin for 3, 6, and 10 minutes as described in Methods and the proteolytic fragments were analyzed by SDS PAGE (Figure 4a). Cleavages occurred only at the tyrosines 585 and 586 as indicated by the observed molecular masses of two chymotryptic fragments. Consistent with this claim, the 72l polypeptide which lacked these tyrosine residue was not digested (lane 9). As predicted, the his-tagged 585/6 polypeptide, generated by a 10-minute chymotryptic digestion of 72k, was retained on Ni-NTA magnetic agarose beads (lane 10). The data showed that under the conditions used, the amino acid residues Y556 and W571 were protected from cleavage by Chymotrypsin and suggested that the RNA binding region was a well-folded domain in which these residues were buried inside the structure. Previously, when extended digestion times were used and polypeptides had been isolated under harsher conditions, a secondary chymotryptic cleavage was observed at Y566. The products of this digestion were unable to form a complex with the SRP RNA [13] thereby supporting the idea that the 545 to 586-region of SRP72 formed a tightly folded domain which, upon destructive cleavage near its center, lost the capacity to bind RNA.

We investigated whether different chymotryptic digestion patterns could be observed when the RNA binding domain of SRP72 formed a complex. Increasing amounts of 72 j polypeptide was incubated with a fixed amount of the human Δ35 [23] or the 5e RNA and formation of complexes was monitored in electrophoretic mobility shift assays (Figures 4d and 4e). Quantitative analysis of the binding reaction (Figure 4c) resulted in an apparent affinity within the previously observed range [13]. Treatment of complexes between 72j and the Δ35 RNA with Chymotrypsin showed that the 585-YY-586 site remained accessible whereas Y566 and W571 remained protected as had been observed in the free polypeptide. Spurious amount of undigested 72j were observed (Figure 4b, lane 4) only at an excess of the Δ35 RNA (corresponding to the concentration used in lane 7 of Figure 4d). These data suggested that larger conformational changes in the SRP72 RNA binding domain were unlikely to occur in the complex, and at least one of the two tyrosine at 585-YY-586 residues (most likely Y586) remained accessible when compared to the free polypeptide.

### The 5e motif is the site of an RNA kink turn

The 5e motif was discovered in an alignment of the SRP RNAs as one of four conserved elements [15,24]. It has remained the least characterized SRP feature. Enzymatic probing revealed that 5e and its surrounding region was remarkably resistant towards digestions by several single-strand specific RNases and also the double-strand specific RNase V1. In particular, the 240-AUC-242 "loop" (Figure 1c) was cleaved at unexpectedly low levels suggesting that the 5e RNA formed a peculiar compact RNase resistant structure [16].

To investigate the possibility of a structural contortion of the 5e RNA motif we adapted a previously developed strategy for the study of DNA bending [25]. The potential kinking of the 5e region was monitored by comparing the electrophoretic mobilities of larger double stranded nucleic acid fragments with the 5e RNA placed either at the center or towards one of the ends. Because a bent or kinked molecule was expected to migrate more slowly in gel electrophoresis compared with a linear fragment of the same size, a mobility difference indicated a deviation from a straight linear molecule [26].

**Figure 5:**
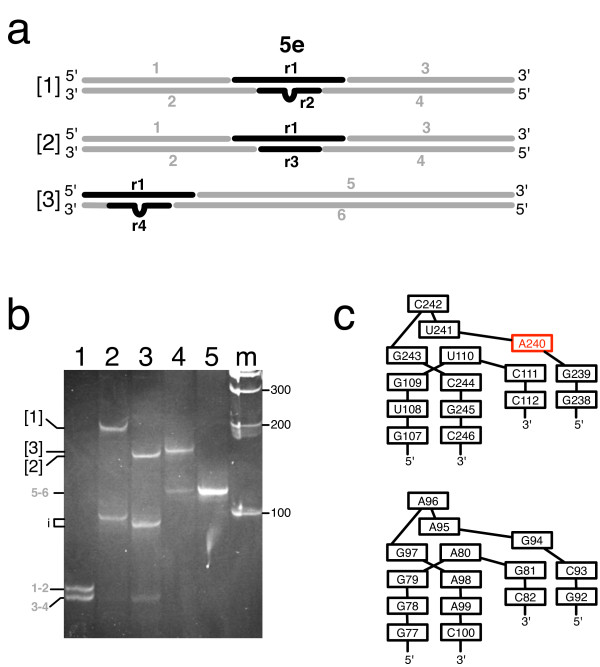
**Analysis of 5e SRP RNA kinking**. a. Polynucleotide fragment assemblies to investigate 5e SRP RNA kinking. In assembly 1, the 5e motif was placed in the middle, residues 240-AUC-242 were deleted in assembly 2, and the 5e motif was placed near the end in assembly 3. Gray and black lines indicate DNA and RNA, respectively. The sequences of the fragments are provided as Additional File 1. b. The annealed polynucleotide fragments were separated by electrophoresis on an eight percent native polyacrylamide gel followed by staining with Ethidium bromide as described in Methods. Lane 1, annealed fragments 1 and 2 (1-2) and annealed fragments 3 and 4 (3-4); lane 2, same as lane 2 with added r1 and r2; lane 3, same as lane 1 with added r1 and r3; lane 4, annealed fragments 5, 6, r1 and r4; lane 5, annealed fragments 5 and 6; lane m, 100 base pair DNA ladder as indicated. The migration distances of the three assemblies, the annealed fragments and some intermediates (i) are labeled on the left. c. Base arrangements in the predicted 5e SRP RNA K-turn (top) in comparison with the Kt-7 kink-turn (bottom)[27].

Using synthetic DNA and RNA oligonucleotides we generated three different assemblies with 10-nucleotide staggered complementary overhangs (Figure 5a and Additional file 1). In the first, the 5e motif was placed in the center. The second assembly also contained RNA at its center but residues 240-AUC-242 were lacking. The 5e RNA motif was placed near the end in assembly 3. The step-wise annealed nucleic acids were separated by electrophoresis on eight percent native polyacrylamide gels followed by staining with Ethidium bromide as described in Methods. Assembly 2 was composed of 151 base pairs and migrated approximately as one would expect for a linear straight double-stranded nucleic acid (Figure 5b, lane 3). In contrast, assembly 1 with the 5e motif at the center travelled considerably slower near the mobility of a 200 base pair double-stranded DNA (lane 2). Assembly 3 with 5e positioned near the end migrated only slightly slower (lane 4) than the straight nucleic acid (lane 3). We concluded that the 5e motif contorted or kinked the RNA by a significant degree, consistent with data showing that this section of the SRP RNA was digested neither by single-strand nor double-strand specific RNases [16].

K-turns, a widespread feature of many medium size and large RNA molecules, have been characterized as being composed of a canonical stem, three unpaired nucleotides, and two adjacent non-canonical GA pairs [27]. Within the 5e motif of the human SRP RNA, the non-canonical GAs were replaced with a conserved G109-C244 and a variable U110-G243 (Figure 5c), but in all other aspects there were striking similarities between 5e and a generic K-turn. Homology modeling of the three-dimensional structure of the 5e section of the human SRP RNA using for comparison the Kt-7 crystal structure from the 23 S rRNA of the *Haloarcula marismortui *ribosome [28] yielded a convincing model lacking atomic clashes (not shown). As in Kt-7, the juxtaposed G109-C244 and U110-G243 were non-canonical. Of particular significance of the 5e model was that the highly conserved A240 residue shown previously to be the key residue for the binding of SRP72 [16] protruded away from the kink. As demonstrated in Figure 5b, the 5e K-turn was stable in a buffer containing 2 mM EDTA as a chelator of divalent cations, but also existed when the EDTA was replaced with 1 mM Magnesium chloride during fragment assembly and electrophoresis (not shown).

### A three-dimensional model of human SRP72 and its RNA binding domain

**Figure 6:**
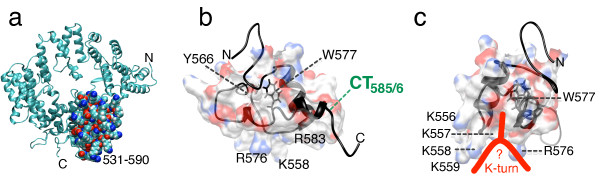
**Three-dimensional model of the 5e RNA binding fragment of human SRP72**. a. Ribbon representation of the three-dimensionaI model of the complete human SRP72 as predicted by I-TASSER [29] and modified as described in Methods. The N- and C-termini are indicated and the atoms of the residues in RNA binding region (532 to 590) are shown as spheres and colored by charge. b. Closeup of the SRP72 RNA binding domain with the identified minimal region required for forming a complex with the 5e SRP RNA in a surface representation. The conserved tryptophan residue located inside the model is drawn in more detail. Shown are the locations of some lysine residues, the prominent chymotryptic cleavage site (CT585/6) and the tyrosine at position 566. c. Same as panel b but viewed at an approximately 90 degree rotation around the y axis revealing a pocket suitable for binding to the kinked 5e SRP RNA (red).

The three-dimensional structure of SRP72 has not been determined experimentally. We therefore used homology modeling of the whole protein and its RNA binding domain in order to gain insights into the mechanism of the binding of RNA by SRP72. The initial SRP72 model was created using the I-TASSER server [29] followed by necessary adjustments to the structure as described in Methods. The final structural model (Figure 6a, and Additional file 2) was consistent with our experimental observations and, at least in part, validated our model. Particularly, the TPR motifs located in the N-terminal region of SRP72 which participated in the formation of a tight complex with SRP68 were predicted correctly [13,30]. Residues 531 to 592, shown as a surface representation in Figure 6a, contained the RNA binding region near the C-terminus of SRP72 as determined by previously published work [12] and the presented data.

The structure of the RNA binding region was predicted to contain two crossed alpha-helices separated by a large loop formed by residues R555 to P572 (Additional file 3). This loop was engaged in maintaining the identified minimal RNA binding domain (E545 to Y586) shown as surface representation in Figures 6b and 6c. Four residues from the lysine cluster were part of alpha-helix 1, but the other lysine residues (K556 to K559, Figure 6c) were located within the loop and exposed. The PDPXRWLPXXER Pfam motif was entirely contained within the second predicted alpha-helix. The space between the loop and helix 2 formed a groove or pocket suitable for binding to the kinked 5e RNA. We speculated that the protuberant A240 residue of 5e engaged within this groove, and predicted that binding was stabilized by residues from the lysine cluster as well as by certain residues of PDPXRWLPXXER. The model suggested that R576 which protruded from the alpha-helix had such a role. In contrast, the chymotryptically inaccessible W577 appeared to be essential for maintaining the structural integrity of the domain. Sequence based (PROSITE) [31] and structure based (VAST) [32] searches with the SRP72 RNA binding domain did not reveal any structural homologues with appreciable similarity. However, an overall similar mode for engaging an RNA K-turn using two regions which embrace the RNA existed in the L30e kink-turn RNA-protein complex [33].

## Conclusions

We presented a model of the RNA binding domain of human SRP72. The results obtained from site-directed mutagenesis and proteolytic digestion experiments were entirely consistent with the predicted structure. We proposed and supported experimentally the idea that the 5e RNA motif formed a stable K-turn which lacked non-canonical G-A pairs and existed independently of divalent cations. All eukaryotic SRP RNAs were likely to use the protruding highly conserved adenosine of the 5e RNA for insertion into a groove or pocket of SRP72. Sequence comparisons indicated that the lysine residues of the human SRP72 were replaced in other organisms with arginines in order to neutralize the negative charges of the RNA. Most likely because of its dual function in binding to the RNA and also to maintain structure, the PDPXRWLPXXER Pfam motif was strictly conserved.

Because even the free RNA was kinked, SRP72 may not be required to stabilize the contorted RNA, but the SRP72 might still alter the bending angle of the RNA.

An approximately 90 degree angle was observed by cryo-electron microscopy in the canine SRP when it was bound to wheat germ ribosomes [10]. The bend or hinge was thought to involve an internal loop at the nucleotide positions 100 and 250 (Figure 1b), but this region of the SRP RNA was poorly defined in previous SRP models. Our data suggested that the 5e motif and not necessarily the neighboring internal loop were responsible for the bent shape of the SRP. These data would also explain the conservation of the 5e motif in the archaea SRPs where SRP68 and SRP72 are absent but the large and small SRP domains are presumably still required to communicate. Further studies will be needed to determine the molecular structure and potential dynamics of the hinge and the precise contribution of the 5e motif to the bent SRP.

## Methods

### Cloning, site-directed mutagenesis, expression and purification of human SRP72 fragments

Bacterial pET based expression plasmids encoding fragments of human SRP72 (untagged 72e, 72f, 72g and 72h) were constructed using as starting material NcoI and BstXI-restricted DNA fragments from a previous study [13]. The N-terminal His-tagged versions were generated by ligating the annealed oligonucleotides CAT GGG ATC GCA TCA CCA TCA CCA TCA CGG GTC TGA AAA CCT GTA TTT CCA GGG CGG AAG and CAT GCT TCC GCC CTG GAA ATA CAG GTT TTC AGA CCC GTG ATG GTG ATG GTG ATG CGA TCC to the NcoI-digested plasmid followed by selection of individual transformants on LB agar plates containing Ampicillin and restriction mapping to identify the correct clone.

Plasmids encoding wild-type or mutant SRP72 fragments with a C-terminal his-tag were generated by PCR using as primers the oligonucleotide GGA CCA TGG TTC GGA AGA AGG GTG GAA AAG TTA CTGG and various other oligonucleotides depending on the desired construct, e.g CGC AAG CTT TCA GTG GTG GTG GTG GTG GTG CTT TCT TCC CCG GTA G. The PCR products were digested with NcoI and HindIII and ligated to the purified NcoI and HindIII-digested 72e vector DNA and clones were selected as described above. To delete or change residues within the lysine-rich cluster, a silent HindIII site was introduced into the coding region for 561-KLP-563 by PCR site-directed mutagenesis [34]. This intermediate vector was digested with NcoI and partially with HindIII, purified and used as a casette to insert and ligate the desired annealed oligonucleotides with compatible NcoI and HindIII ends. The coding regions of all construct were confirmed using commercial services.

For the expression of the his-tagged SRP72 fragments plasmids were transformed into the competent cells of the *Escherichia coli *strain Rosetta (pLysS). Transformants were selected by an overnight incubation at 37°C on LB agar plates containing Ampicillin and Chloramphenicol [16]. Colonies were used to inoculate 15 ml cultures of LB with antibiotics followed by shaking at 37C until the A280 reached approximately 0.2. Protein expression was initiated by adding IPTG to a final concentration of 0.5 mM and shaking was continued for 5 hrs. at room temperature. Cells were pelleted by centrifugation and stored at -70°C.

The frozen pellet from 2.5 ml of cell culture was resuspended in 2.5 ml of ice-cold lysis buffer (50 mM Na-phosphate, pH 7.5, 500 mM NaCl, 10 mM Imidazole, 5 mM 2-Mercapto ethanol, 1 M urea) supplemented with 20 ul of protease inhibitor cocktail equivalent to 1/75th of one EDTA-free complete mini protease tablet (Roche). The cell suspension was sonicated (Fisher Scientific, model 300) 6 times for 15 sec each at a setting of 35 percent with 1 minute interruptions during which the sample was placed on ice. The lysate was subjected to centrifugation in a Beckman ultracentrifuge for 1 hr at 50,000 rpm and 4 C (Beckman TLA-100.3 rotor) and the supernatant was added to a 200 ul suspension of Ni-NTA Superflow beads (Quiagen) which had been pre-equilibrated in lysis buffer. The sample was mixed by slow rotation for 1 hr at 4°C and transferred to 0.5 ml mini-columns (Evergreen Scientific). After the column had settled by gravity flow, the beads were washed with 1 ml of lysis buffer. The his-tagged bound proteins were collected by washing in two steps with 150 μl of lysis buffer containing 150 mM and then 300 mM Imidazole. Sample aliquots were subjected to electrophoresis on 12.5% polyacrylamide SDS Tricine gels followed by staining of the polypeptides with Coomassie blue with known amounts of Lysozyme for reference to calculate protein concentrations from a standard curve.

### In vitro synthesis of SRP RNAs

Full-length human SRP RNA (hR) or its fragment from the large SRP domain (Δ35 RNA) were synthesized by run-off transcription of DraI- or BamHI-restricted plasmids with T7 RNA polymerase [23]. The construction of the plasmid template for the 5e RNA and its in vitro transcription was performed as described [16].

### Magnetic bead binding assays

Binding of the his-tagged polypeptides to the 5e or Δ35 SRP RNA was measured using Ni-NTA magnetic agarose beads (Quiagen) essentially as described [16]. Bead-bound polypeptides and SRP RNAs were separated in the magnetic field, washed, and eluted with the buffer containing 250 mM Imidazole. Samples were analyzed by electrophoresis on 12.5% polyacrylamide Tricine-SDS gels, followed by staining of the polypeptides with Coomassie blue. After the destaining with 40% Methanol, 10% Acetic acid, the gel was washed once with water and incubated for 10 minutes with 0.1 mg/ml buffered Ethidium bromide. The RNA was visualized by placing the gel onto a UV-transilluminator (Bio-Rad) and a picture was recorded. The number of pixels in each band was measured using NIH Image software [35].

### Chymotryptic accessibility and RNA protection assays

Polypeptides at a concentration of 0.25 to 0.3 mg/ml were incubated in a volume of 10 μl lysis buffer containing 300 mM Imidazole (from the purification step) with 0.3 μg of alpha-Chymotrypsin (Sigma, type VI) for up to 10 minutes at ambient temperature. The digestion was terminated by adding 6 μl of SDS-loading buffer (100 mM Tris-HCl, pH 6.8, 4% SDS, 20% glycerol) and the samples were analyzed by electrophoresis on 12.5% Tricine-SDS polyacrylamide gels followed by staining with Coomassie blue.

For the identification of his-tagged chymotryptic fragments, the digested samples were adjusted to 100 μl and a final concentration of 50 mM Na-phosphate, pH 7.5, 500 mM NaCl and 0.1% Tween 20 (binding buffer). 15 μl of a Ni-NTA magnetic bead suspension (Quiagen) were added follwed by occasional mixing with a pipette at room-temperature for 1 hr. The beads were separated from the unbound polypeptides by placing the samples into a magnetic field, the beads were washed with 50 μl of binding buffer and the bound polypeptides were eluted with the same buffer but containing 250 mM Imidazole. Samples were analyzed by SDS PAGE and the gels stained with Coomassie blue.

To measure the ability of the RNA to protect protein from the digestion by Chymotrypsin, the purified 72j polypeptide was mixed at room temperature in 12.5 μl of 50 mM Tris-HCl, 300 mM KOAc, 5 mM MgCl_2_, pH 7.9 with increasing amounts of in vitro transcribed Δ35 RNA. The samples were incubated for 10 minute at 37°C and then placed at room temperature. 0.3 μg of Chymotrypsin were added and complexes were digested for 5 minutes. The digestion was terminated by adding 4 μl of Tris-acetate EDTA buffer followed by SDS PAGE as described above.

### Electrophoretic mobility shift assay

For the formation of complexes between the 72j polypeptide and the SRP RNA, 0.25 μg of 5e or Δ35 RNA were mixed with variable amounts of 72j protein in 10 μl of 50 mM Tris-HC, l pH 7.9, 300 mM KOAc, 5 mM MgCl_2 _and 16% glycerol. Samples were incubated for 10 minutes at 37°C and separated by electrophoresis at room temperature on a 6% poyacrylamide gel (Acryl/Bisacryl 19:1) in 20 mM HEPES, 0.1 mM EDTA, pH 8.3 at 15 mA for 2 hrs. A picture was recorded after staining of the RNAs with buffer containing 0.1 mg/mL Ethidium bromide and placing the gel onto a UV-transilluminator. The number of pixels in each band was measured using NIH Image software [35].

### RNA bending assay

400 μmole of each complementary nucleic acid were annealed in 400 μl of TE containing 100 mM NaCl. Samples were placed in a boiling water bath and allowed to gradually cool to room temperature during a time period of about 30 minutes. The annealed nucleic acids were extracted with an equal volume of phenol/chloroform and the aqueous supernatant of the centrifugation was mixed with 15 μl of 5 M NaCl and 3 volumes of ethanol. The nucleic acids were incubated at -70°C for 20 min and the precipitate was collected by centrifugation, washed with 300 μl 80 percent ethanol and dissolved in 100 μl of TE.

4 μmole of the annealed DNA fragments were mixed with equivalent molar amounts of synthetic oligoribonucleotides in 20 μL of 50 mM Tris-HCl, pH 7.5, 25 mM NaCl, with or without 1 mM MgCl_2_. Samples were allowed to gradually cool from 70°C to 4°C within approximately 30 minutes. 5 μl aliquots of the samples were subjected to electrophoresis at 4°C and 160 V using a 8% acrylamide gel (Acryl/Bisacryl 19:1) in TBE or in TB with 1 mM MgCl_2 _until the Bromophenol blue dye had migrated 10 cm. The gel was stained for 10 minutes with 0.1 mg/ml Ethidium bromide and the nucleic acids were visualized using a UV-transilluminator (Bio-Rad) to record a picture.

The sequences of the nucleic acids are provided in Additional file 1.

### Molecular modelling of human SRP72

The three-dimensional model of the complete amino acid sequence of human SRP72 was created by the I-Tasser server [29]. From the proposed models we selected the one with the best scores and representing the structure which was similar to other proteins containing tetratricopeptide repeats (TPRs). Because not all the clashes were removed by the program, we rebuild the loop composed of residues 476 to 486 using Swiss-PdbViewer [36]. The atomic coordinates of the edited structure in pdb format are provided as Additional files 2 and 3 and were used for the creation of Figure 6 which shows one possible spatial organization of the human SRP72 molecule and its RNA-binding region. Molecular graphics images were produced using VMD [37] for Figure 6a, and the UCSF Chimera package [38] for Figures 6b and 6c.

## Authors' contributions

EI purified wild-type and mutant polypeptides, transcribed the RNAs, and carried out all binding assays. AI created the three-dimensional model of the human SRP72 and participated in the assessing the spatial relationship between RNA and its interaction with SRP72. CZ conceived the study, generated the mutant plasmids, carried out the RNA bending experiments, and drafted the manuscript. All authors read and approved the final manuscript.

## Supplementary Material

Additional file 1 **Sequences of the synthetic polynulceotides used to investigate kinking of the 5e motif in human SRP RNA**. Oligonucleotides are numbered as in Figure 5a. Ribonucleic acid residues are shown underlined. Click here for file

Additional file 2 **Atomic coordinates of the predicted structure of human SRP72 in pdb format**. Click here for file

Additional file 3 **Predicted atomic coordinates of human SRP72's RNA binding domain**. Click here for file
