# Supplementary material for: Identification of amino acid residues in protein SRP72 required for binding to a kinked 5e motif of the human signal recognition particle RNA
Source: BMC Mol Biol. 2010 Nov 13;11:83. doi: 10.1186/1471-2199-11-83 (PMC2995471; doi:10.1186/1471-2199-11-83)
Supplement: Additional file 1 — Sequences of the synthetic polynulceotides used to investigate kinking of the 5e motif in human SRP RNA. Oligonucleotides are numbered as in Figure 5a. Ribonucleic acid residues are shown underlined. [file 1471-2199-11-83-S1.PDF]

deoxyoligonucleotide 1 (56-mer)

GAATTCACGC GTAGATCTGC TAGCATCGAT CCATGGACTA GTCTCGAGTT TAAAGA

deoxyoligonucleotide 2 (66-mer)

CAGCTGGATA TCTTTAAACT CGAGACTAGT CCATGGATCG ATGCTAGCAG ATCTACGCGT
GAATTC

deoxyoligonucleotide 3 (56-mer)

CTTCGCGAAA TATTGGTACC CCATGGAATC GAGGGATCCT CTAGAGTTAA CGTCGA

deoxyoligonucleotide 4 (66-mer)

TCGACGTTAA CTCTAGAGGA TCCCTCGATT CCATGGGGTA CCAATATTTC GCGAAGGCCT
CCCGGG

deoxyoligonucleotide 5 (112-mer)

GAATTCACGC GTAGATCTGC TAGCATCGAT CCATGGACTA GTCTCGAGTT TAAAGACTTC
GCGAAATATT GGTACCCCAT GGAATCGAGG GATCCTCTAG AGTTAACGTC GA

deoxyoligonucleotide 6 (122-mer)

TCGACGTTAA CTCTAGAGGA TCCCTCGATT CCATGGGGTA CCAATATTTC GCGAAGTCTT
TAAACTCGAG ACTAGTCCAT GGATCGATGC TAGCAGATCT ACGCGTGAAT TCGCCTCCCG
GG

oligonucleotide r1 (39-mer)

UAUCCAGCUG GAUCGGGUGU CCCACUAAGU CCCGGGAGG

oligonucleotide r2 (23-mer)

ACUUAGUGGG AUCACACCCG AUC

oligonucleotide r3 (20-mer)

ACUUAGUGGG ACACCCGAUC

hybrid oligonucleotide r4 (33-mer)

ACUUAGUGGG AUCACACCCG AUCCAGCTGG ATA
